# Knockdown of MRPL35 promotes cell apoptosis and inhibits cell proliferation in non-small-cell lung cancer

**DOI:** 10.1186/s12890-023-02677-0

**Published:** 2023-12-13

**Authors:** Chengling Zhao, Lei Chen, Zhixin Jin, Haitao Liu, Chao Ma, Hangtian Zhou, Lingling Xu, Sihui Zhou, Yan Shi, Wei Li, Yuqing Chen, Chengli Dou, Xiaojing Wang

**Affiliations:** 1 Anhui Province Key Laboratory of Clinical and Preclinical Research in Respiratory Disease, Department of Pulmonary and Critical Care Medicine, First Affiliated Hospital of Bengbu Medical College, Bengbu, 233004 China; 2 Clinical Research Center for Respiratory Disease (Tumor) in Anhui Province, Bengbu, 233004 China; 3 Molecular Diagnosis Center, First Affiliated Hospital of Bengbu Medical College, Bengbu, 233004 China

**Keywords:** MRPL35, NSCLC, Proliferation, Apoptosis, p53

## Abstract

**Background:**

Non-small cell lung cancer (NSCLC) is a major pathological type of lung cancer. However, its pathogenesis remains largely unclear. MRPL35 is a regulatory subunit of the mitoribosome, which can regulate the assembly of cytochrome c oxidases and plays an important role in the occurrence of NSCLC.

**Methods:**

The expression of MRPL35 in NSCLC was detected by tissue microarray and immunohistochemistry. H1299 cells were infected with lentivirus to knockdown MRPL35, and the cells were subjected to crystal violet staining to assess the results of colony formation assays. A549 cells were infected by lentiviral particles-expressing shMRPL35 or shControl, and then subcutaneously injected into nude mice. Tumorigenesis in mice was detected by in vivo imaging. The potential pathway of MRPL35 in NSCLC was assessed by Western blotting.

**Results:**

MRPL35 was over-expressed in NSCLC tissue compared to para-cancerous and normal tissues. Knockdown of MRPL35 suppressed cell proliferation and decreased NSCLC progression both in vitro and in vivo. The possible molecular mechanisms were also clarified, which indicated that MRPL35 could be involved in cell apoptosis and proliferation by modulating the expression levels of CDK1, BIRC5, CHEK1, STMN1 and MCM2. Knockdown of MRPL35 activated p53 signaling pathway and inhibited cell cycle regulation.

**Conclusions:**

The oncogenic role of MRPL35 in NSCLC was potentially mediated through the cell cycle regulatory genes such as BIRC5, STMN1, CDK1, CHEK1 and MCM2, as well as activation of P53 signaling pathway.

**Supplementary Information:**

The online version contains supplementary material available at 10.1186/s12890-023-02677-0.

## Introduction

Lung cancer is the most common cause of cancer-related death worldwide, which includes two main subtypes: non-small cell lung cancer (NSCLC) and small cell lung cancer (SCLC), with incidence rates of 85% and 15%, respectively [1]. According to the pathological features, NSCLC can be further classified into the following subtypes: large cell lung cancer, lung adenocarcinoma (LUAD), and lung squamous cell carcinoma (LUSC) [2]. NSCLC is a devastating disease with the five-year survival rate of 16% [3]. The most common treatment for lung cancer is surgery, but it can confer a poor prognosis. Thus, research on the molecular mechanism of NSCLC is needed for the early diagnosis, targeted treatment and prevention of NSCLC.

Mitochondria are essential for cellular energy production and biosynthetic reactions, which consume approximately 90% of total oxygen in mammalian cells. They play key roles in cellular homeostasis, apoptosis regulation and energy metabolism [4–6]. Mitochondrial ribosomal proteins (MRPs) share homology with mitochondria-specific proteins and prokaryotic ribosomal proteins. After synthesis of MRPs in the cytoplasm, they are transported to the mitochondria from the cytoplasmic matrix to realize mitochondrial ribosome assembly and mitochondrial protein translation [7]. Mitochondrial dysfunction can promote cancer progression through different mechanisms. Deregulation of cellular energetic metabolism is one of the hallmarks of cancer. It has been suggested that mitochondrial dysfunction is associated with deregulated cellular energetics [8]. Human mitoribosome is composed of a large 39S subunit and a small 28S subunit [9].

Recent studies have shown that in addition to cellular energy production and mitochondrial protein synthesis, some mitotic ribosomal proteins are also involved in the cellular processes, including apoptosis regulation, cell cycle progression, and mitochondrial homeostasis [10]. MRPL35 participates in the formation of the central protrusion region of the large (54S) mitotic ribosomal subunit, however, its function has not been fully investigated yet. A previous research demonstrated that MRPL35 could regulate the assembly of cytochrome c oxidases by coordinating the interaction of COX1 with specific assembly proteins [11].

Furthermore, several studies have suggested that the expression levels of MRP genes may vary in different types of cancer, and these variations are associated with various clinical features of certain types of cancer [12]. For instance, MRPL35 expression is related on a prolonged survival of glioblastoma patients [13]. The expression level of MRPL35 is also up-regulated in colorectal cancer cells [14]. In hepatocellular carcinoma cells, a high expression level of MRPS23 can stimulate cell proliferation, and confers to a poor prognosis [15]. We previously found that MRPL7, MRPL40, MRPL15, and MRPL22 are the markers of mitochondrial translation and mitochondrial biogenesis, which may contribute to the progression of breast cancer [16].

In this study, we assessed the expression level and functional significance of MRPL35 in NSCLC. The results demonstrated that MRPL35 was over-expressed in NSCLC tissues. Knockdown of MRPL35 suppressed cell proliferation and decreased NSCLC progression both in vitro and in vivo. The possible molecular mechanisms were also clarified to indicate that MRPL35 could be involved in the cell apoptosis and proliferation by modulating the expression levels of CDK1, BIRC5, CHEK1, STMN1 and MCM2. Our findings revealed that MRPL35 could play a crucial role in the development of NSCLC, and it could be a novel therapeutic target for this disease.

## Results

### MRPL35 is over-expressed in NSCLC tissue

**Fig. 1:**
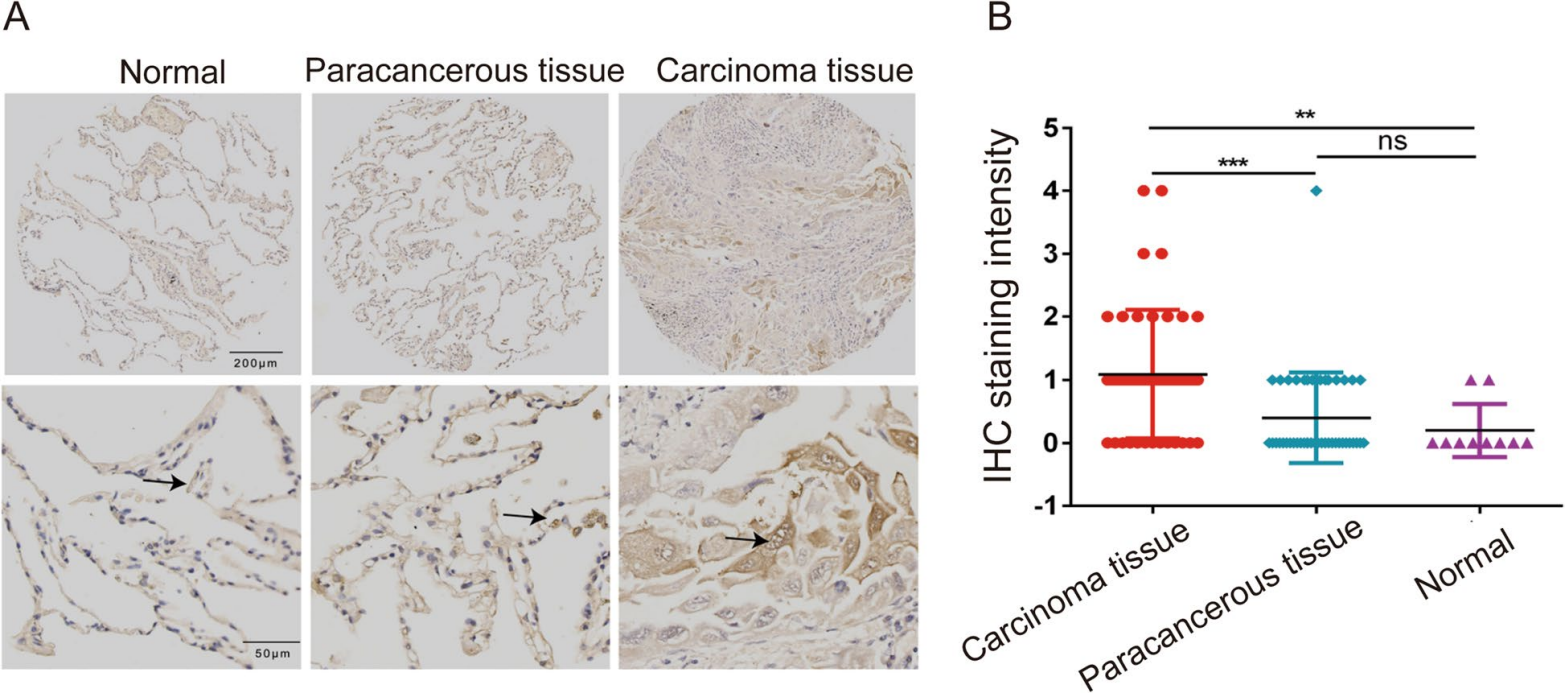
MRPL35 was over-expressed in NSCLC tissues. **A** IHC results of the expression level of MRPL35 in normal tissue, para-cancerous tissue, and NSCLC tissue. **B** Statistical analysis results of IHC staining intensity

A tissue microarray was obtained from US Biomax Inc. (Rockville, MD, USA) to analyse the expression level of MRPL35. The tissue microarray included 45 NSCLC tissues, 45 para-cancerous tissues, and 10 normal tissues. The immunohistochemistry (IHC) results indicated that MRPL35 was over-expressed in NSCLC tissue compared to para-cancerous and normal tissues (Fig. 1A). Based on the staining sites, we calculated the MRPL35 protein expression scores (range = 0–12) by multiplying the positive and intensity score. The results of statistical analysis further confirmed that MRPL35 was over-expressed in cancer tissues (Fig. 1B).

### Knockdown of MRPL35 inhibits tumor growth in vitro

**Fig. 2:**
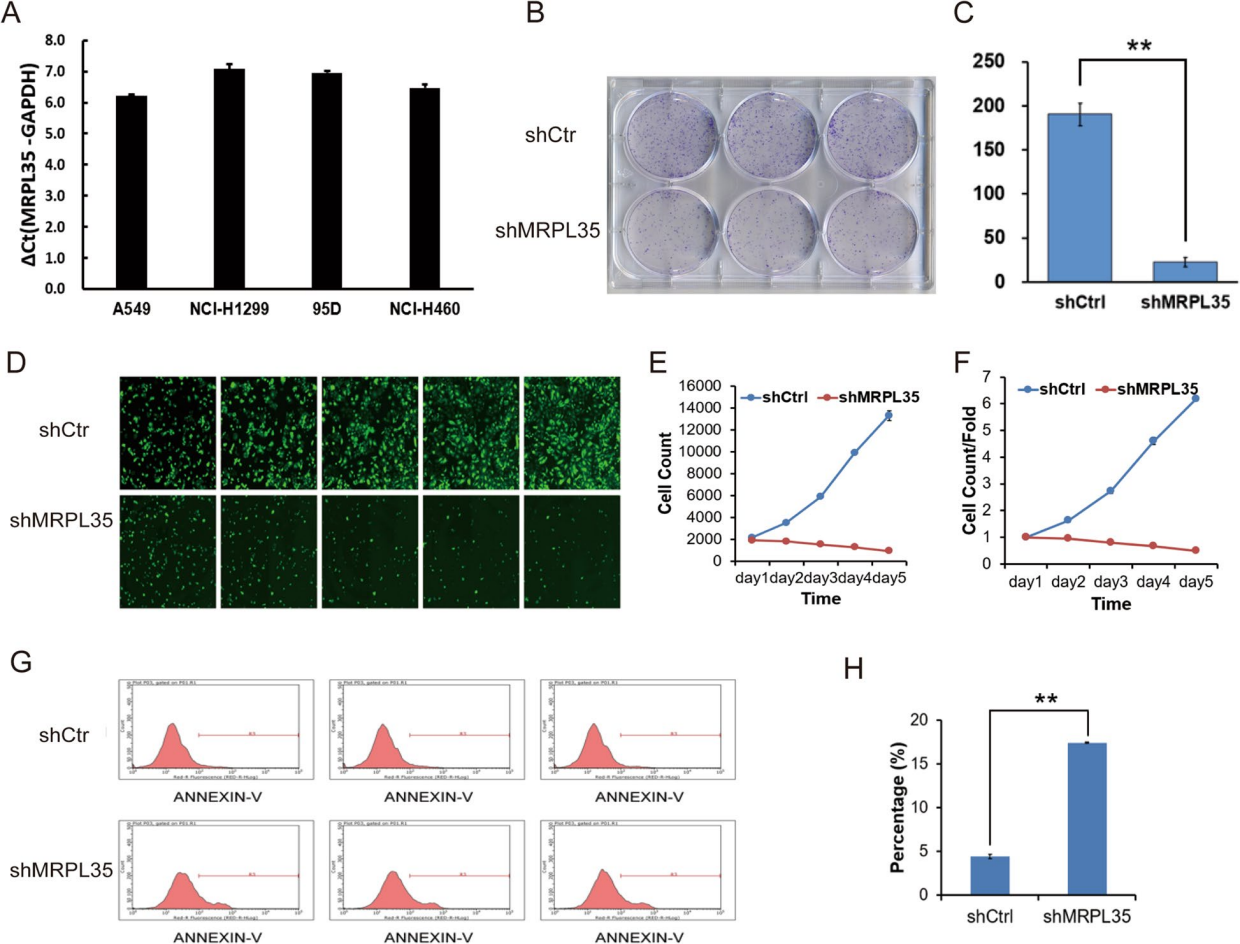
MRPL35 knockdown promotes cell apoptosis and inhibits cell proliferation. **A** The mRNA level of MRPL35 in A549, NCI-H1299, 95-D, and NCI-H460 cells was detected by qPCR. **B** Colony formation assays. **C** Statistical analysis results of colony formation assays. **D** Cell proliferation was assessed by celigo cell count assays. **E**–**F** Statistical analysis results of celigo cell count assays. **G** Cell apoptosis in control and MRPL35-knockdown groups was detected by flow cytometry. **H** Statistical analysis results of flow cytometry

To further explore the roles of MRPL35 in the occurrence and development of NSCLC, the expression level of MRPL35 was detected in a variety of NSCLC cell lines. First, the mRNA level of MRPL35 in cells was examined by quantitative PCR (qPCR), and the data indicated that MRPL35 was highly expressed in A549, NCI-H1299, 95-D, and NCI-H460 cells (Fig. 2A). Subsequently, H1299 cells were infected with lentivirus to knockdown MRPL35 (Supplementary Fig1), and the cells were subjected to crystal violet staining to assess the results of colony formation assays (Fig. 2B and C). The results demonstrated that the number of colonies was decreased after knockdown of MRPL35, implying that MRPL35 expression was significantly correlated with the clonogenic ability of NCI-H1299 cells (Fig. 2B and C). To further verify the relationship between MRPL35 expression and cell proliferation, the cells were grown in a 96-well plate after lentiviral vector transduction for 3 days, and continuously monitored for another 5 days. The results showed that NCI-H1299 cell proliferation was remarkably inhibited after knockdown of MRPL35 (Fig. 2D). Furthermore, the number of cells was quantitatively analyzed, and the results demonstrated that MRPL35 expression was marked associated with the proliferation of NCI-H1299 cells (Fig. 2E and F). In addition, flow cytometric analysis was conducted to assess the effects of knockdown of MPPL35 gene on NSCLC cell apoptosis. After knockdown of MRPL35, the apoptosis of NCI-H1299 cells was significantly increased in the experimental group, suggesting that MRPL35 expression was markedly related to the apoptosis of NCI-H1299 cells (Fig. 2G and H).

### Knockdown of MRPL35 promotes cell apoptosis and suppresses cell proliferation in vivo

**Fig. 3:**
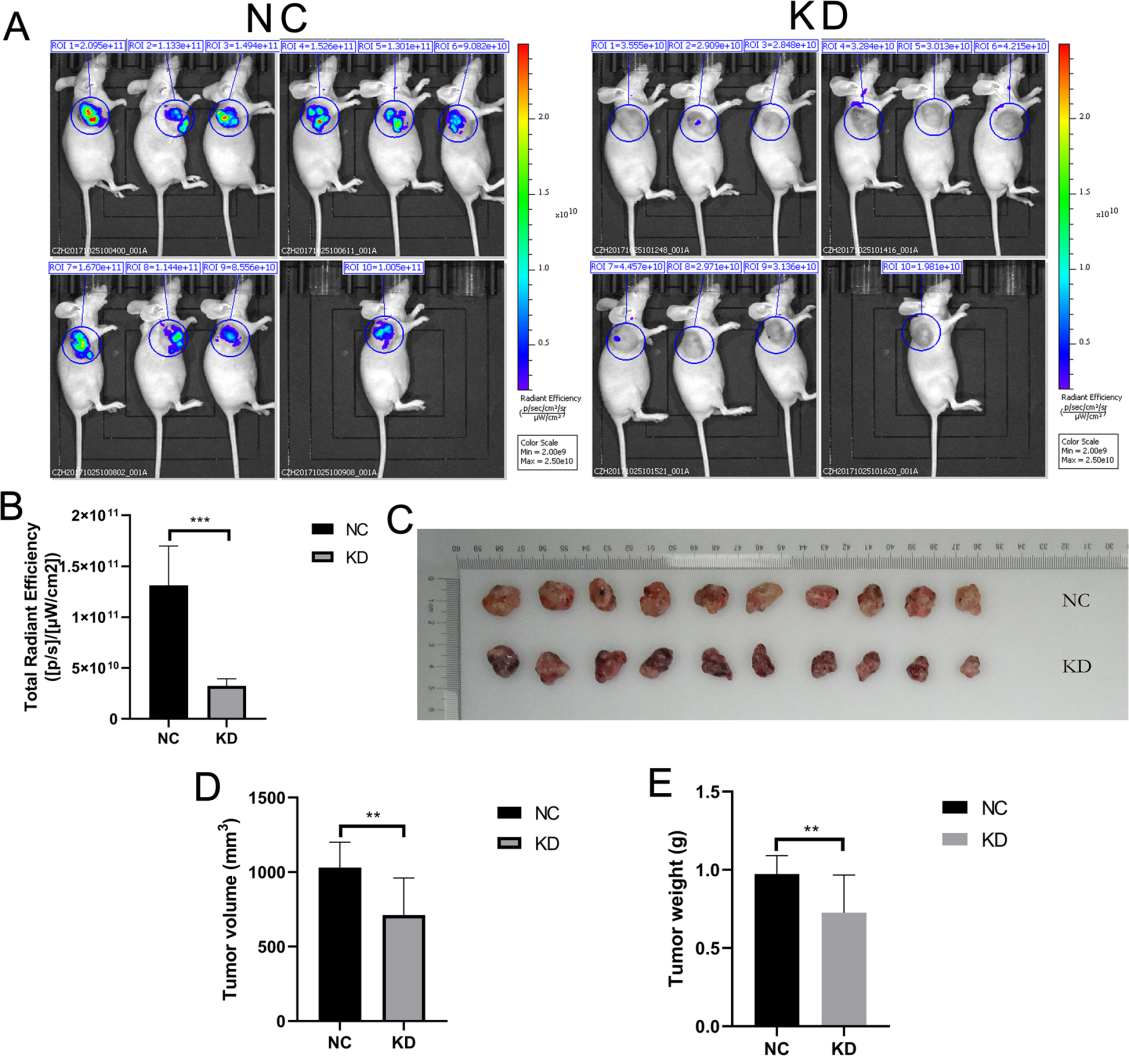
Knockdown of MRPL35 suppresses tumor growth in tumor-bearing mice. **A** In vivo imaging of tumor-bearing mice in control group and MRPL35-knockdown group. **B** Statistical analysis results of total radiant efficiency. **C** Tumor size was measured in NC and MRPL35-knockdown groups. **D**-**E** Measurement of the volume and weight of tumors

To examine the oncogenic activity of MRPL35 in vivo, A549 cells were infected by lentiviral particles-expressing shMRPL35 or shControl, and then subcutaneously injected into nude mice. After cell injection for 20 days, tumorigenesis in mice was detected by in vivo imaging. Compared to the negative control (NC) group, knockdown of MRPL35 significantly decreased the tumorigenesis ability of mice receiving A549 cells (Fig. 3A and B). To further verify this result, the tumor-bearing mice were executed one month after A549 cell injection and tumor were removed (Fig. 3C). Subsequently, the volume and weight of tumor were measured, which were significantly reduced in MRPL35-knockdown group (Fig. 3D and E).

### MRPL35 silencing activates p53 signaling pathway and inhibits cell-cycle in NSCLC cells

**Fig. 4:**
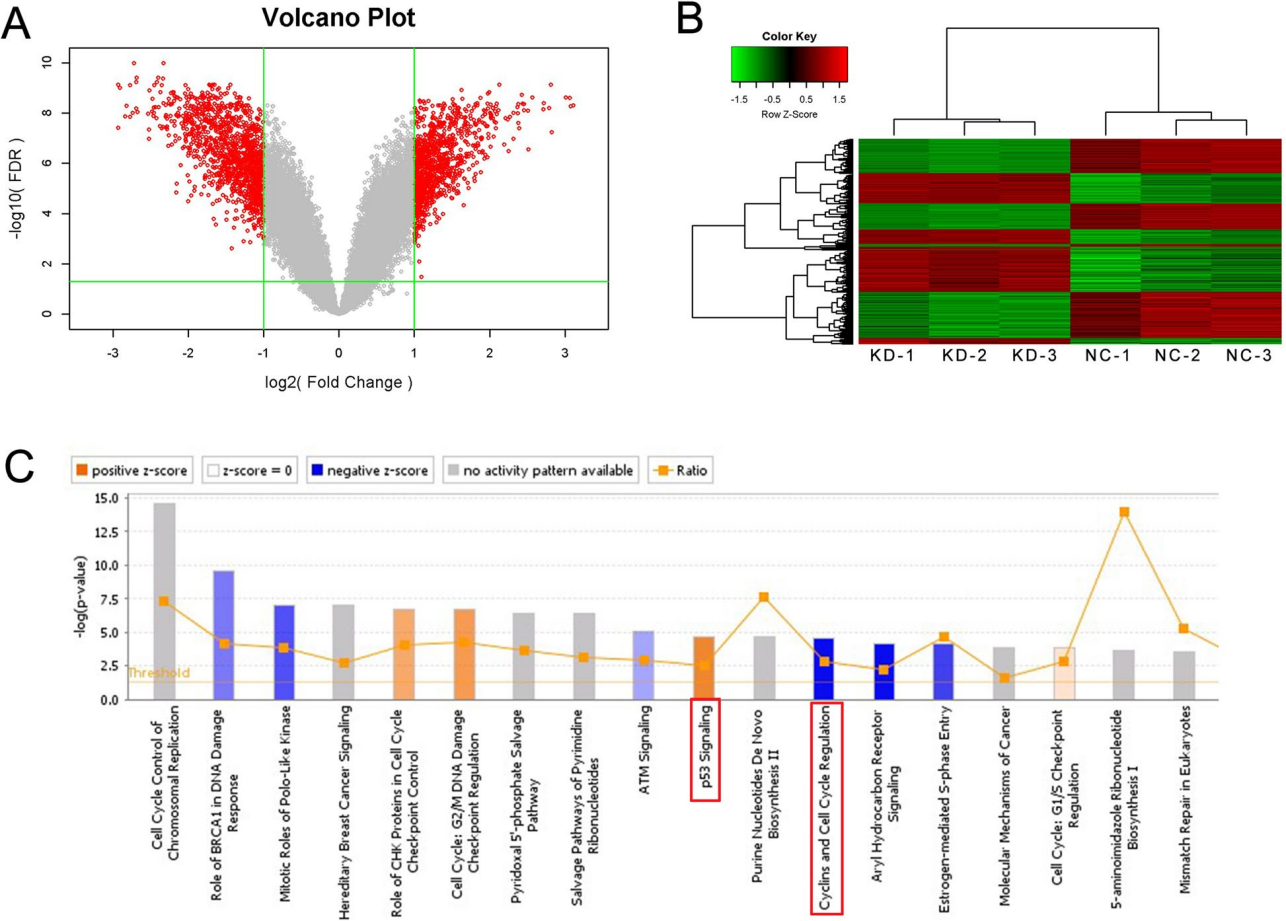
Transcriptome sequencing of NC group and MRPL35-knockdown group. **A**  Differentially expressed genes are illustrated using a volcano plot. B Heatmap showing the results of hierarchical clustering. C Canonical pathway enrichment analysis of differentially expressed genes. The red box marks the activation of the p53 signaling pathway and the inhibition of cyclins and cell cycle regulation

To further explore the molecular mechanism underlying the inhibitory and inductive effects of MRPL35 of cell proliferation and apoptosis, respectively, the transcriptome sequencing of NCI-H1299 cell lines was analyzed after knockdown of MRPL35. Compared to the control group, 693 and 786 genes were up-regulated and down-regulated, respectively, after knockdown of MRPL35. The volcano plot (Fig. 4A) was drawn based on the differences in gene expression levels between the two groups of samples, as well as false discovery rate (FDR). Hierarchical clustering of MRPL35-knockdown and NC samples (Fig. 4B) was carried out according to these differentially expressed genes (DEGs). Furthermore, the data of ingenuity pathway analysis (IPA) indicated that the DEGs were remarkably enriched in the classical pathway. In addition, p53 signaling pathway was remarkably activated, while the levels of cyclins and cell-cycle regulation were markedly suppressed (Fig. 4C). Altogether, these findings indicate that knockdown of MRPL35 can activate p53 signaling pathway and suppress the levels of cyclins and cell-cycle regulation.

### MRPL35 is involved in cell apoptosis and proliferation by modulating the expression levels of CDK1, BIRC5, CHEK1, STMN1 and MCM2

**Fig. 5:**
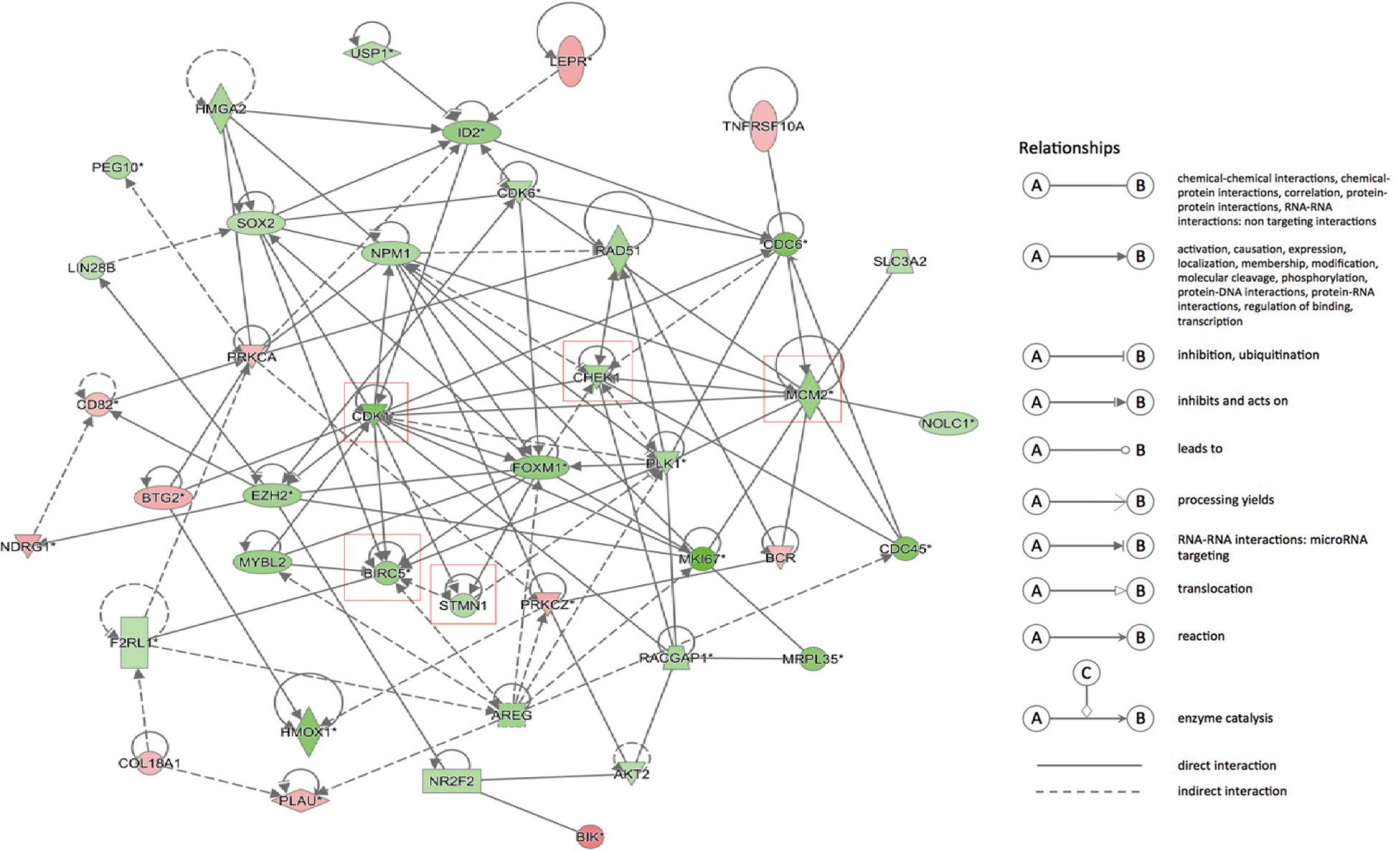
Interaction network of DEGs related to the cell proliferation and apoptosis. Red indicates the up-regulated genes after knockdown of MRPL35, green denotes the down-regulated genes, and different shapes represent different protein classifications. The red box marks the down-regulated genes related to cell proliferation and apoptosis after knockdown of MRPL35

**Fig. 6:**
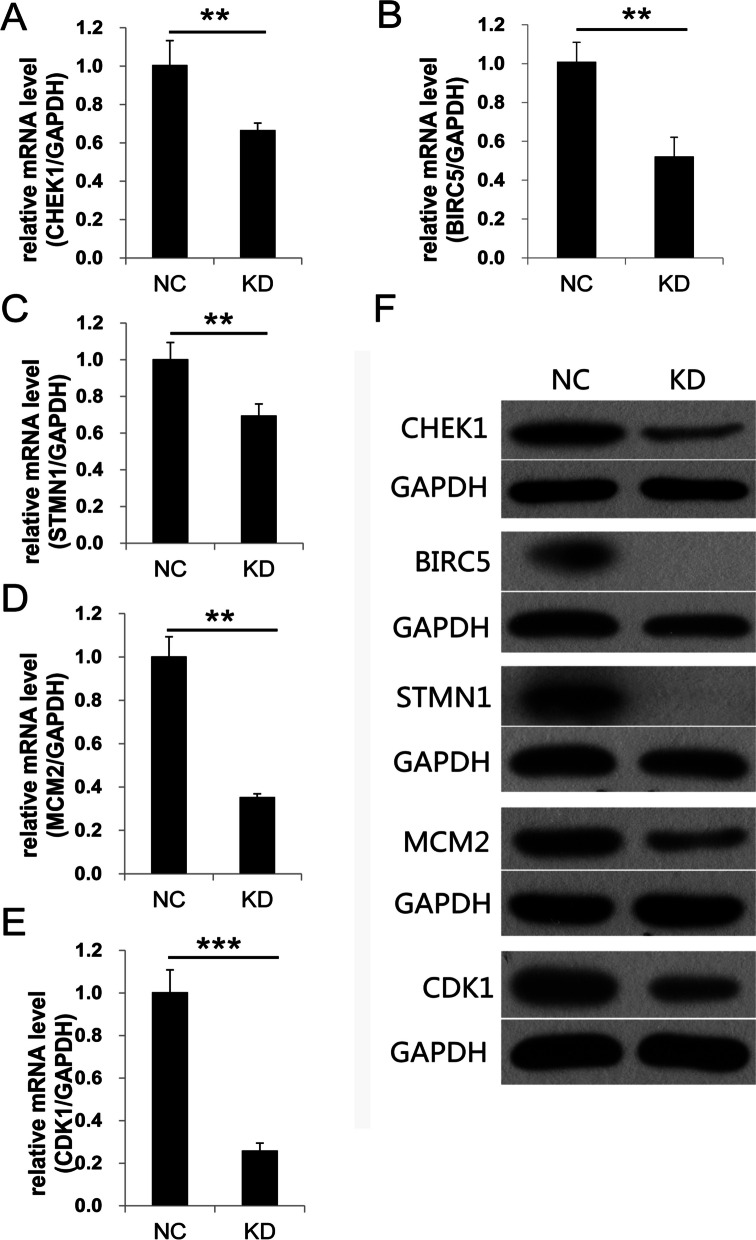
Knockdown of MRPL35 results in the down-regulation of CHEK1, BIRC5, STMN1, MCM2 and CDK1. **A**-**E** The mRNA levels of CHEK1, BIRC5, STMN1, MCM2 and CDK1. **F** The protein levels of CHEK1, BIRC5, STMN1, MCM2 and CDK1. Full-length blots gels are presented in Supplementary Fig. 2

The up-regulated or down-regulated genes related to cell proliferation or apoptosis obtained by transcriptome sequencing were analyzed by bioinformatics to explore the protein–protein interaction (PPI) network. The results of bioinformatics analysis revealed that MRPL35 could play a crucial role in NSCLC by regulating the expression levels of BIRC5, STMN1, CDK1, CHEK1 and MCM2 (Fig. 5). Moreover, the mRNA and protein levels of these five genes were evaluated by qPCR and Western blotting (Fig. 6A-F). Full-length blots/gels are presented in Supplementary Fig. 2. The results demonstrated that the mRNA and protein levels of BIRC5, STMN1, CDK1, CHEK1 and MCM2 were noticeably down-regulated in MRPL35-knockdown group compared to those in NC group. Collectively, the above-mentioned findings suggest that MRPL35 expression may play a vital role in modulating the expression levels of genes (BIRC5, STMN1, CDK1, CHEK1 and MCM2) related to cell proliferation and apoptosis.

## Discussion

The present study revealed that MRPL35 was over-expressed in NSCLC tissue compared to para-cancerous and normal tissues. Besides, knockdown of MRPL35 attenuated NSCLC progression both in vitro and in vivo by promoting cell apoptosis and suppressing cell proliferation. The results of classical pathway analysis showed that p53 signaling pathway was significantly activated, while the levels of cyclins and cell-cycle regulation were markedly inhibited, which characterized by the down-regulated expression of related genes such as CDK1, BIRC5, CHEK1, STMN11 and MCM. Altogether, these findings indicated that MRPL35 could play a major role in the development of NSCLC, and might serve as a therapeutic target for this disease.

Mitochondria are associated with tumorigenesis, however, this association still remains elusive [17, 18]. Mitochondrial dysfunction could promote cancer progression through different mechanisms [8]. MRPL35 participates in the translation of mitochondrial proteins and assembly of mitoribosomes [7]. Several studies have demonstrated that MRPL35 is involved in cell apoptosis, proliferation and metastasis [19, 20]. MRPL35 participates in the formation of the central protrusion region of the large mitotic ribosomal subunit (54S), which plays a vital role in coordinating the interaction of COX1 with specific assembly proteins [11]. Overexpression of MRPL41 and MRPS36 was proved to contribute to p53 stability, induce apoptosis, and cause delay in cell-cycle progression [21, 22], which were consistent with our results.

The expression levels of MRP genes could vary in different types of cancer, and those levels could be associated with various clinical features of some types of cancer [12]. It has been reported that glioblastoma, colorectal cancer, HCC, and breast cancer are related to MRP genes, while no study has concentrated on the relationship between MRP genes and lung cancer.

The down-regulated expression of MRPL35 can increase reactive oxygen species (ROS) levels, correlating with DNA damage [23]. Increased ROS levels may lead to more extensive and irreparable cell damage via DNA oxidation [24, 25]. Besides, DNA damage contributes to the cell apoptosis by stabilizing p53 [26, 27]. The p53 gene was first described in 1979, and it is known as the first tumor suppressor gene [28]. P53 is involved in DNA repair, cell senescence, cell-cycle arrest, apoptosis, and other cellular processes to inhibit tumorigenesis [29].

During G1 phase, the activity of cyclin-dependent kinase (CDK) increases, thus promoting DNA replication and initiating G1-S phase transition. CDK can further increase its own activity through a positive-feedback loop, which leads to cell division via genome-wide transcriptional regulation [30]. CDK1 and CDK2 play a vital role in triggering the transition from G1 to S phase and from G2 to M phase. Aberrant activation and inhibition of CDK1/2 can affect the progression of cancer [31]. CDK1 is indispensable because it can substitute other CDKs and promote cell-cycle progression [32]. A previous research demonstrated that CDK1 is critical for coupling cell proliferation with protein synthesis via regulation of translation machinery [33]. Up-regulation of p53 inhibited the expression levels of CDK1 and cyclins B1/D1, thus causing a cell cycle progression delay at G2/M phase [34]. Checkpoint kinase 1 (Chk1, CHEKh1) is a critical regulator of cell cycle arrest in G2 phase [35], which can be activated by phosphorylation of Ser345 and Ser317 to respond to DNA damage [36]. A sustained phosphorylation of Chk1 is strongly associated with the phosphorylation of p53, and p53-dependent Chk1 phosphorylation is needed for maintaining the prolonged cell-cycle arrest in G2 phase [37].

The BIRC5 (survivin) is one of the genes located on chromosome 17q, and it is the smallest inhibitor of apoptosis (IAP) protein that responsible for the modulation of cell cycle and inhibition of apoptosis [38]. P53 can regulate the expression levels of cellular homeostasis-related genes, including PTEN, FOXO1 and BIRC5 [39]. Stathmin-1 (STMN1), or OP18, is an oncogene that encodes a cytosolic phosphoprotein of approximately 18 kDa, which has been proved to induce differentiation, proliferation and migration of cancerous cells, and confer a poor prognosis [40–42]. Previous research has demonstrated that STMN1 is down-regulated by p53, and regulates cell-cycle arrest [43]. It has been reported that p53 can inhibit STMN1 expression level, and regulate cell-cycle arrest in G2 or M phase [44].

Besides, MCM2 belongs to the minichromosome maintenance (MCM) protein complex containing 6 highly conserved proteins (MCM2-7) [45]. MCM2-7 proteins play regulatory roles in cell proliferation, and the expression levels of MCM2-7 are regulated during cellular senescence and cell-cycle arrest [46, 47]. It was pointed out that mRNAs could degrade MCM2-7 Ros level in aged cells, and they could be synthesized via the activation of p53 signaling pathway. Hence, the downregulated expression of MRPL35 can increase ROS production, thus leading to DNA damage. DNA damage stabilizes p53 and acts on CDK1, BIRC5, CHEK1, STMN1 and MCM2 to regulate the processes of cell cycle and apoptosis.

Although the molecular mechanisms underlying the promotion of cell apoptosis and inhibition of cell proliferation after knockdown of MRPL35 still remain elusive, our study reveals that MRPL35 expression is closely associated with the progression of NSCLC, and it can serve as be a new therapeutic target for this disease.

## Materials and methods

### Cell culture

Human NSCLC cell lines (H1299 and A549) were purchased from the cell line bank of Bioon (Shanghai, China). The culture medium was Roswell Park Memorial Institute (RPMI)-1640 containing 10% FBS and penicillin–streptomycin. Cells were cultured in a 5% CO_2_ humidified incubator at 37 °C, and the medium was refreshed every 2–3 days. Upon reaching a confluence of 70–80%, 0.25% trypsin solution was used to harvest the cells. Before cell culture, the poly HIPE scaffolds were immersed in ethanol (75%) for 12 h, rinsed thrice with phosphate-buffered saline, and exposed to ultraviolet light for 6 h.

### Production of lentivirus by transient transfection

The linearized vector was obtained via restriction enzyme digestion. The target tablets were prepared by primer annealing. The designed primers were added into enzyme digestion sites at both ends. After annealing, the primers were not linearized, and the two ends of the cloning vector contained the same restriction sites. The reaction system was prepared with the linearized carrier and annealing products, and the final product was directly transformed. The monoclonal antibodies on the plate were chosen for polymerase chain reaction (PCR), followed by sequencing of the positive clones. The correct bacterial clones were expanded, cultured and isolated to achieve high-quality plasmids for viral packaging. Then, the high-purity plasmids were used to co-transfect 293 T cells. After transfection for 48–72 h, the cells were harvested.

Accumulated viral supernatants were gathered and subjected to concentration. At the logarithmic growth stage, H1299 and A549 cells were trypsinized, and the cell suspension (3–5 × 10^4^ cells/ml) was prepared. The cells were then inoculated into a 6-well plate. The optimal amount of infectious D614 and G614 viruses was added, and the infection efficiency was assessed. The lentiviral knockdown system used the GV115 plasmid, which carries EGFP fluorescence, in order to trace the location of tumors in vivo.

### qPCR assays

Extraction of total RNA from the samples was conducted using TRIzol reagent. cDNA synthesis was performed with 500 ng RNA. 500 ng totalRNA was reverse transcribed to obtain cDNA with cDNA Synthesis Kit (Vazyme, R212-01) according to the manufacturer’s protocol. Subsequently, qPCR was conducted using the SYBR Premix Ex Taq kit (Takara, Japan) with cDNA template. The mRNA expression levels were analyzed using the 2^−ΔΔCt^ method. The primer sequences used in the study were as follows: GAPDH, 5′- TGACTTCAACAGCGACACCCA-3′ (F) and 5′-CACCCTGTTGCTGTAGCCAAA-3′ (R); MRPL35, 5′-TTGGCATCTTCAACCTACCGC-3′(F) and 5′-GGAGGAAACAACTGGTGTCTGA-3′ (R).

### Western blotting

Briefly, RIPA buffer was used to isolate the total protein, and the obtained protein specimens were analyzed by BCA Protein Assay kit (Solarbio Science & Technology). Next, the proteins were separated through SDS-PAGE, and then transferred onto PVDF membranes. Non-fat milk was used to block the membranes for 1 h, followed by overnight incubation with primary antibodies at 4 °C: MRPL35 (1:500, SIGMA, HPA043482) β-actin(1:5000, Santa Cruz, sc-69879); CHEK1 (1:500, CST, #2360); BIRC5 (1:1000, CST, #2808); STMN1 (1:1000, CST, #3352); MCM2 (1:2500, Abcam, ab108935); CDK1 (1:500, CST, #9116). Then, the membranes were washed with TBST and incubated with secondary antibodies at 37 ℃ for 1 h: goat anti-mouse IgG (1:2000, SantaCruz, sc2005); rabbit IgG (1:2000, CST, #7074); mouse IgG (1:2000, CST, #7076). Finally, the blots were examined using an ECL detection kit (Pierce Biotechnology, MA, USA).

### Flow Cytometry and IHC

Cell lines were cultured separately for control and experimental groups. Cells were collected, washed, and maintained in single-cell suspension. Cell suspensions were appropriately fluorescently stained to assess the expression levels of annexin V. Samples were analyzed using a flow cytometer to collect cell data, including fluorescent signals.

Lung cancer samples were fixed and embedded to prepare sections. Immunohistochemistry staining was performed on tissue sections using MRPL35 antibody (1:500, SIGMA, HPA043482). The tissue sections were washed with PBS and then incubated with horseradish peroxidase-labeled secondary antibodies for 30 min. Following another PBS wash, the sections were subjected to 3,3'-diaminobenzidine (DAB) staining to visualize the binding of antibodies to the target proteins. Subsequently, the stained sections underwent dehydration and clearing processes before being coverslipped.

## In vivo imaging

A549 cells were infected by lentiviral particles-expressing shMRPL35 or shControl, prepared into cell suspension (2E + 7/mL), and then subcutaneously injected into nude mice. After cell injection for 20 days, tumorigenesis in mice was detected by in vivo imaging. D-Luciferin (15 mg/mL) was injected intraperitoneally at a dose of 10 ul/g for 10 mintues. Then, animals were anesthetized by intraperitoneal injection of 0.7% pentobarbital sodium at the dosage of 10 ul/g a few minutes after in vivo live imaging. Fuorescence examination and data processing were carried out.

### Tumor formation analysis

The study protocol was reviewed and approved by the Ethics Review Committee at Bengbu Medical College. After trypsin digestion of NSCLC cells at the logarithmic growth stage, the cells were re-suspended with a complete medium. A disposable sterile syringe was used to subcutaneously inject cells into the mice. After 5–20 days, the tumor formation was assessed, and the volume and weight of tumor were measured. After 28 days of subcutaneous injection, the mice were euthanized by an excessive injection of 2% pentobarbital sodium, and cervical dislocation was performed to confirm the cell death. Then, photographs were captured using a digital camera. The tumor was taken out using the conventional surgical instruments. Finally, the removed tumor was weighed, and the data were recorded.

### Celigo cell count assays

The infected cells (1000 cells/well) were inoculated into a 6-well plate for about 14 day, and the medium was refreshed every 3 days. After that, the cells were fixed with paraformaldehyde (4%) for 40 min, followed by staining with crystal violet (0.1%) for 4 min. The manual counting of colonies was performed.

### Colony formation assays

The infected cells (1000 cells/well) were inoculated into a 6-well plate, and the medium was refreshed every 3 days. After two weeks, paraformaldehyde fixation and crystal violet staining were conducted as described above. Manual colony counting was then performed.

### Statistical analysis

Statistical tests were performed using the GraphPad Prism v6.0 (GraphPad Software Inc., CA, USA). The two-tailed Student’s t-test was utilized for comparing the differences between groups. All assays were repeated 3 times.

### Supplementary Information

**Additional file 1: Supplementary Figure 1.** Detection of the knockdown efficiency of H1299 cells transfected with virus by qPCR and Western blotting. After shRNA lentivirus infection, the expression of MRPL35 in NCI-H1299 cells was inhibited (P<0.05) at the mRNA level. **Supplementary Fig 2. **The protein levels of MRPL35, CHEK1, BIRC5, STMN1, MCM2 and CDK1 between NC and KD groups.

## Data Availability

The datasets generated and/or analysed during the current study are available in the Gene Expression Omnibus (GEO) repository. https://www.ncbi.nlm.nih.gov/geo/query/acc.cgi?acc=GSE225959.The data that support the findings of this study are available from the corresponding author upon request.
