# Supplementary material for: Knockdown of MRPL35 promotes cell apoptosis and inhibits cell proliferation in non-small-cell lung cancer
Source: BMC Pulm Med. 2023 Dec 13;23:507. doi: 10.1186/s12890-023-02677-0 (PMC10720070; doi:10.1186/s12890-023-02677-0)
Supplement: Supplementary file 1 — Additional file 1: Supplementary Figure 1. Detection of the knockdown efficiency of H1299 cells transfected with virus by qPCR and Western blotting. After shRNA lentivirus infection, the expression of MRPL35 in NCI-H1299 cells was inhibited (P<0.05) at the mRNA level. Supplementary Fig 2. The protein levels of MRPL35, CHEK1, BIRC5, STMN1, MCM2 and CDK1 between NC and KD groups. [file 12890_2023_2677_MOESM1_ESM.docx]

**Supplementary Fig1**


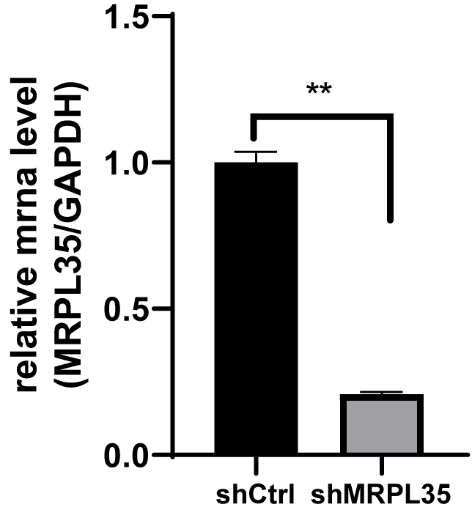


Supplementary Figure 1: Detection of the knockdown efficiency of H1299 cells transfected with virus by qPCR and Western blotting. After shRNA lentivirus infection, the expression of MRPL35 in NCI-H1299 cells was inhibited (P<0.05) at the mRNA level.

**Supplementary Fig 2**


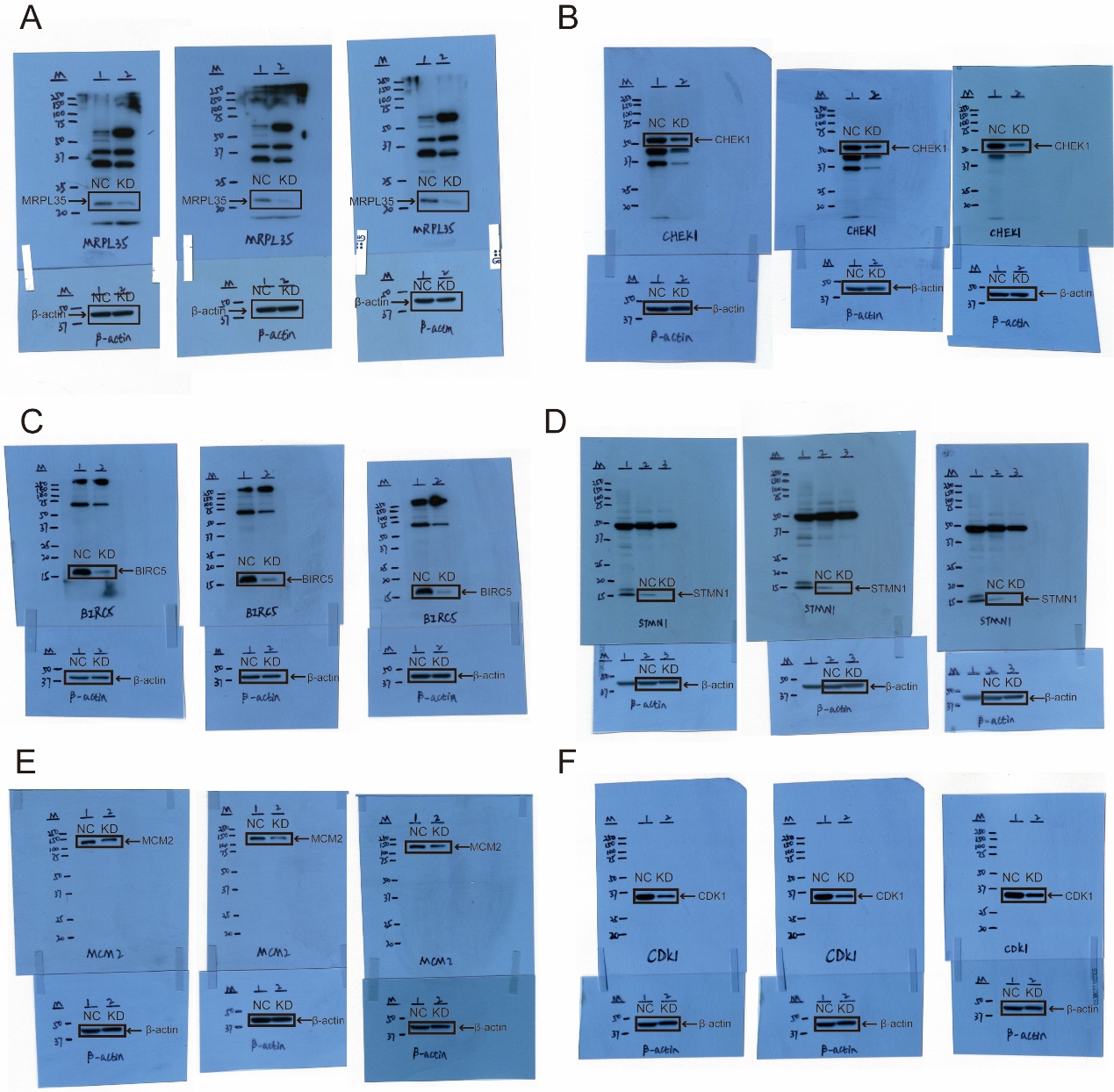


The protein levels of MRPL35, CHEK1, BIRC5, STMN1, MCM2 and CDK1 between NC and KD groups.
